# Pure Platelet and Leukocyte–Platelet-Rich Plasma for Regenerative Medicine in Orthopedics—Time- and Preparation-Dependent Release of Growth Factors and Effects on Synovial Fibroblasts: A Comparative Analysis

**DOI:** 10.3390/ijms24021512

**Published:** 2023-01-12

**Authors:** Erminia Mariani, Lia Pulsatelli, Luca Cattini, Paolo Dolzani, Elisa Assirelli, Annarita Cenacchi, Alessandro Di Martino, Carla Renata Arciola, Giuseppe Filardo

**Affiliations:** 1 Department of Medical and Surgical Sciences, Alma Mater Studiorum-Università di Bologna, 40138 Bologna, Italy; 2 Laboratory of Immunorheumatology and Tissue Regeneration, IRCCS Istituto Ortopedico Rizzoli, 40136 Bologna, Italy; 3 Medicine and Rheumatology Unit, IRCCS Istituto Ortopedico Rizzoli, 40136 Bologna, Italy; 4 Single Metropolitan Transfusion Service, AUSL Bo, 40136 Bologna, Italy; 5 Clinica Ortopedica e Traumatologica 2, IRCCS Istituto Ortopedico Rizzoli, 40136 Bologna, Italy; 6 Laboratorio di Patologia delle Infezioni Associate all’Impianto, Laboratorio di Immunoreumatologia e Rigenerazione Tissutale, IRCCS Istituto Ortopedico Rizzoli, 40136 Bologna, Italy; 7 Applied and Translational Research Center, IRCCS Istituto Ortopedico Rizzoli, 40136 Bologna, Italy

**Keywords:** growth factors, chemokines, release kinetics, platelet-rich plasma, tissue repair, regenerative medicine, synovial fibroblasts, orthopedics, osteoarthritis

## Abstract

Intra-articular injections of autologous platelet concentrates are considered capable to enhance the healing of cartilage lesions, alleviate joint inflammation, and relieve other musculoskeletal pathological conditions. The aim of this study was to analyze the soluble fractions obtained from platelet-rich plasma (pure- and leukocyte-PRP) to compare time- and preparation-dependent modifications of growth factor concentrations and the supporting activity of the two preparations on synovial fibroblast growth and hyaluronic acid (HA) production in vitro. The release kinetics of FGF-2, SDF-1, VEGF, HGF, EGF, PD GF-AB/BB, IGF-1, VCAM-1, and TGF-β isoforms were followed up to 168 h after PRP activation, and their amounts were determined by multiplex-beads immunoassay. Synovial cell growth and supernatant HA production were respectively analyzed by Alamar Blue assay and ELISA. Time-dependent modifications grouped molecules in three peculiar patterns: one reaching the highest concentrations within 18 h and decreasing afterwards, another progressively increasing up to 168 h, and the last peaking at the central time points. Synovial fibroblast growth in response to L-PRP and P-PRP revealed differences over time and among added concentrations. Both preparations displayed a preserved supporting capacity of HA synthesis.

## 1. Introduction

Platelet concentrates are an up-and-coming tool for regenerative therapy in orthopedics. Early inflammatory osteoarthritis (OA) of the knee and hip as well as tendinopathies appear to benefit from the therapeutic use of autologous platelet-rich preparations. Indeed, platelets release a rich pool of growth factors (GFs), which can promote the repair of bones, tendons, and joint tissues of the human musculoskeletal system. The complex network of GFs, such as transforming growth factor (TGF), platelet-derived growth factor (PDGF), insulin growth factor (IGF), fibroblast growth factor (FGF), and hepatocyte growth factor (HGF), is known to play a regulatory role in musculoskeletal and joint tissues homeostasis and to be involved in their repair processes [1,2]. GFs together with many other bioactive molecules (cytokines and chemokines) are pre-packaged in the alpha granules, which represent the platelet storage units. Platelets are natural sources of signaling molecules with paracrine effects. The application of platelet concentrates [3,4,5], mostly represented by platelet-rich plasma (PRP) and platelet-rich fibrin (PRF), provides an appealing solution to promote healing of cartilage lesions of traumatic origin and to treat joint inflammatory processes, such as osteoarthritis (OA) and other musculoskeletal pathological conditions (such as muscle injuries, tendinopathies, and intervertebral disc degeneration) [6,7,8,9], both in old patients and in young athletes. Noticeably, septic arthritis also appears to benefit from bactericidal activity of PRP treatment [10], as recently reported using an equine infectious arthritis model [11].

The progressive diffusion of PRP use is mainly due to the characteristics of biocompatibility, quick and easy preparation, low cost and, last but not least, acceptance both by the patient for the minimally invasive route of administration and the clinician for the immunogenic safety due to the autologous blood derivation.

Over the past decade, the therapeutic efficacy of PRP for the hard and soft tissue wound-healing process has been widely reported, suggesting that PRP may exploit different repair mechanisms in a variety of pathophysiological processes (including hemostasis, inflammation, infection, angiogenesis, and the synthesis of the extracellular matrix). PRP appears to be able to protect synoviocytes and chondrocytes of the joint from inflammation and to modify the biological microenvironment at different points of the disease process, thus offering the opportunity to interfere with the insidious self-perpetuating mechanisms of chronic diseases (such as OA) [9,10,11,12].

Despite the promising preliminary results reported for clinical applications, PRPs are obtained by heterogeneous preparations that still lack consensus in terms of both technical procedures and biological aspects (centrifugation speed, platelet concentration, presence of leukocytes, and activator substances). All these variables may influence biological factor release mechanisms and cell/tissue responses, ultimately affecting clinical outcomes [13,14,15,16,17].

At present, L-PRF (leukocyte-PRF) is a commonly used platelet concentrate as it is easy to obtain. It is suitable to be applied as such in the dental field, spread for the treatment of chronic wounds, leg ulcers, and diabetic foot [18], or applied to the ocular surface for the treatment of corneal lesions [19]. However, it is not suitable for injection. Hence, the use of L-PRP, which activated by CaCl_2_ undergoes a slower gelation and therefore lends itself to be injected into closed cavities. Furthermore, the majority of studies on the use of platelet rich GFs have described only the outcome at the cellular and tissue level without addressing the kinetics of GF release from the fibrin scaffold [9].

The purpose of this study was to analyze the soluble fraction released by the activated clots obtained from the two most described PRP preparations, one characterized by an enrichment of pure platelets (pure platelet-rich plasma, P-PRP) and the other by the concomitant presence of platelets and leukocytes (leucocyte–platelet-rich plasma, L-PRP), to compare the time- and preparation-dependent modifications of GF concentrations between these two blood derivatives. For this purpose, we focused on various GFs displaying important effects on joint tissue regeneration, and we followed the in vitro time-dependent delivery of epidermal growth factor (EGF), fibroblast growth factor (FGF)-2, HGF, insulin growth factor (IGF)-1, platelet-derived growth factor (PDGF) AB/BB, stromal-cell-derived factor (SDF)-1, vascular-cell-adhesion molecule (V-CAM)-1, vascular endothelial growth factor (VEGF), and transforming growth factor (TGF)-β1, β2, β3 up to 168 h after PRP activation.

Furthermore, the present study aimed at testing the supporting activity of the two preparations on the function of synovial fibroblasts by evaluating the in vitro cell growth and hyaluronic acid (HA) production.

## 2. Results

### 2.1. Platelet and White Blood Cell Concentrations

Platelets were slightly increased in P-PRP compared with the peripheral blood, and they were more concentrated in L-PRP than in P-PRP. White blood cells (WBCs) next to the lowest limit of detection in the P-PRP preparation, maintained concentrations overlapping those of the peripheral blood in L-PRP (Table 1). Platelets and WBCs in PPP were below the linearity detection limits of the analyzer (not shown).

### 2.2. Growth Factor Concentrations: Time- and Preparation-Dependent Modifications

**Figure 1:**
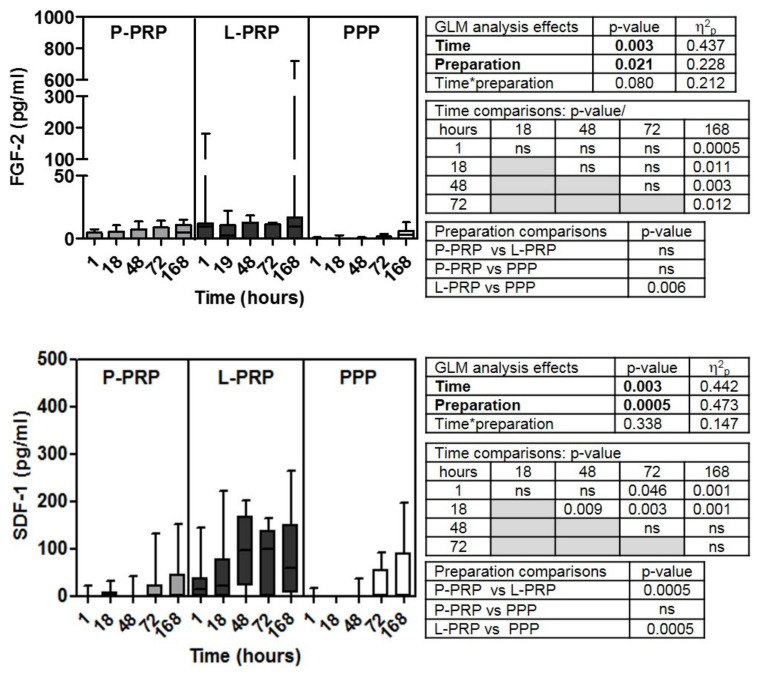
Concentrations of FGF-2 and SDF-1/CXCL-12 molecules in P-PRP, L-PRP, and PPP. Results are reported as medians (bars), 25th and 75th percentiles (boxes), and minimum to maximum values (whiskers); *p* value significances are reported in tables on the right side of each graph, as determined by general linear mode statistical analysis (bold); * variable association; ns, not significant.

**Figure 2:**
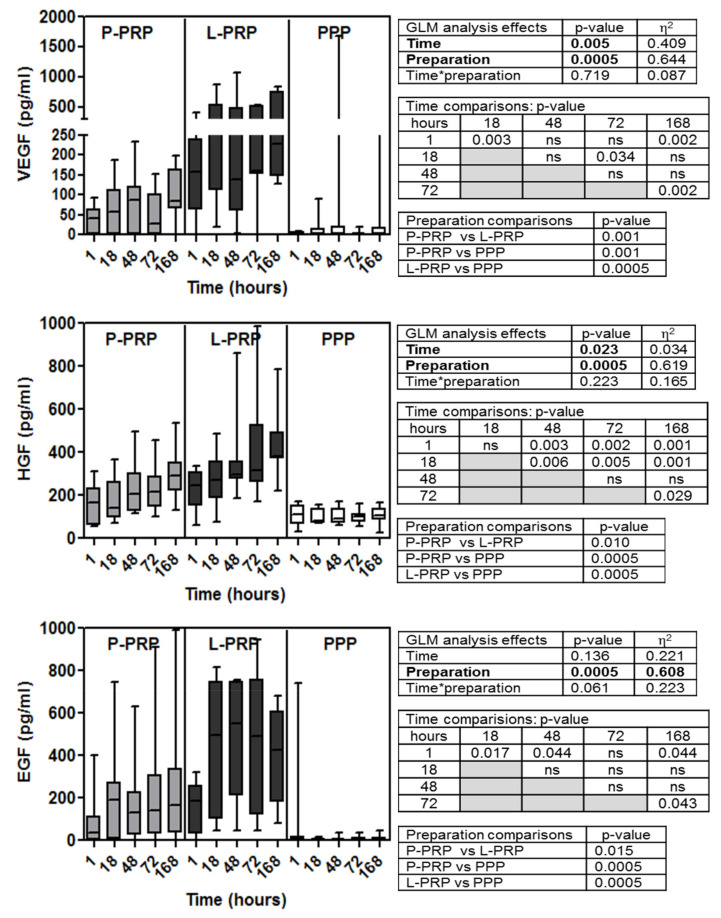
Concentrations of VEGF, HGF and EGF molecules in P-PRP, L-PRP, and PPP. Results are reported as medians (bars), 25th and 75th percentiles (boxes), and minimum to maximum values (whiskers); *p* value significances are reported in tables on the right side of each graph, as determined by general linear mode statistical analysis (bold); * variable association; ns, not significant.

**Figure 3:**
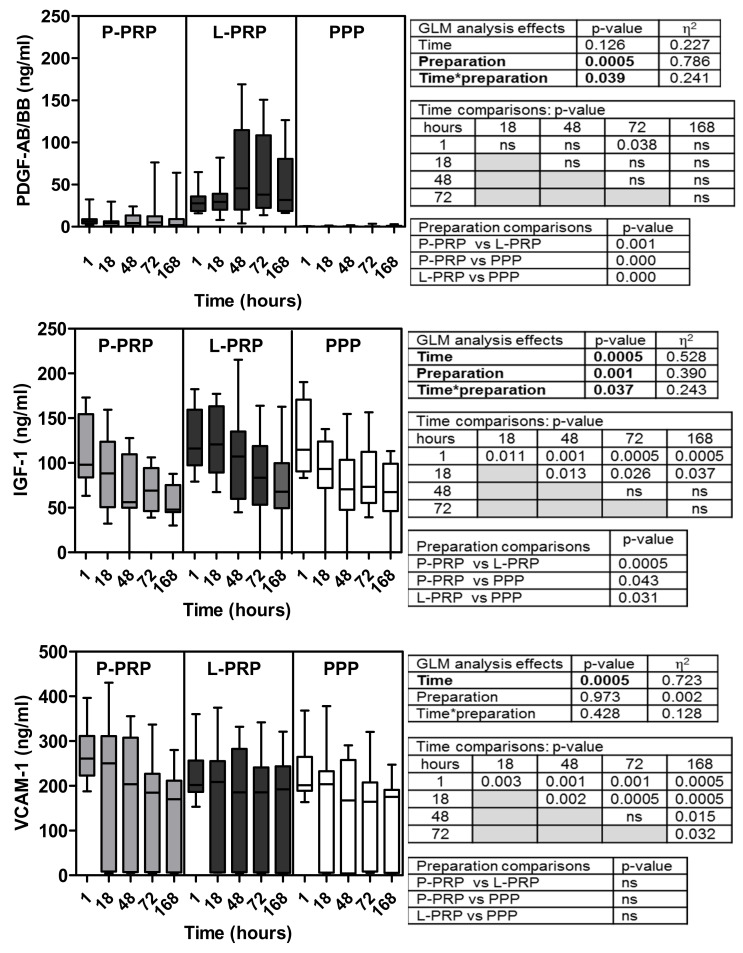
Concentrations of PDGF-AB/BB, IGF-1, and V-CAM-1 molecules in P-PRP, L-PRP, and PPP. Results are reported as medians (bars), 25th and 75th percentiles (boxes), and minimum to maximum values (whiskers); *p* value significances are reported in tables on the right side of each graph, as determined by general linear mode statistical analysis (bold); * variable association; ns, not significant.

**Figure 4:**
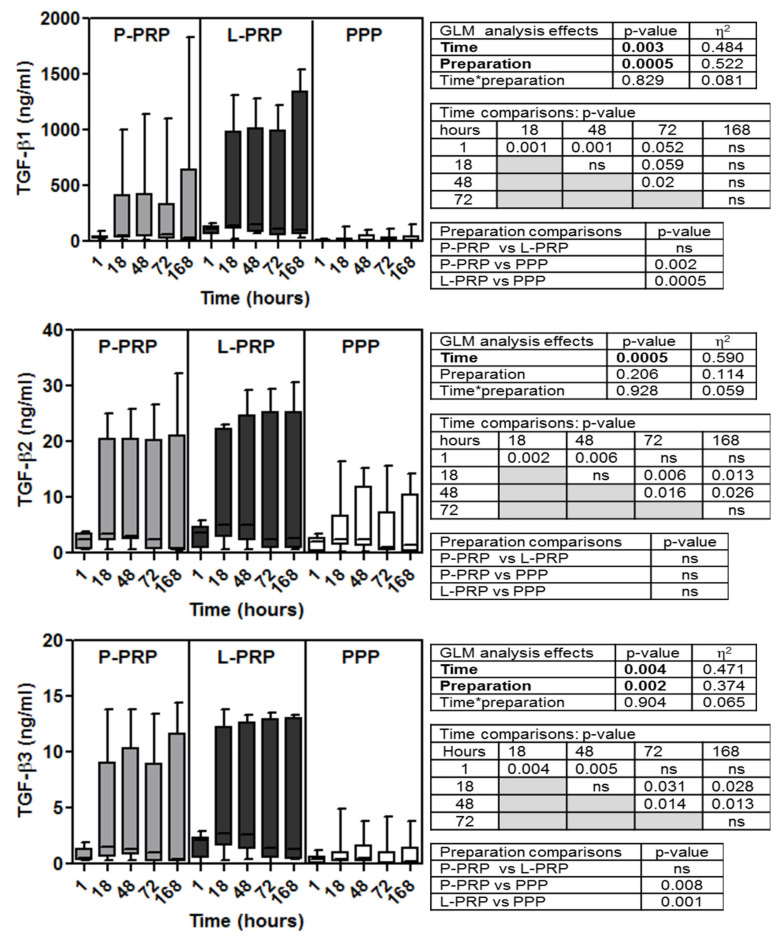
Concentrations of TGF-β1, β2, and β3 in molecules in P-PRP, L-PRP, and PPP. Results are reported as medians (bars), 25th and 75th percentiles (boxes), and minimum to maximum values (whiskers); *p* value significances are reported in tables on the right side of each graph, as determined by general linear mode statistical analysis (bold); * variable association; ns, not significant.

Soluble factor data are reported in Figure 1, Figure 2, Figure 3 and Figure 4 for increasing concentrations from pico to nanograms per unit of volume (excluded TGF-β2 and three that are cumulatively reported with the β1 isoform)). Inter-donor coefficients of variability that referred to the time-dependent release of each growth factor were reported in Table S1 (Supplementary File).

PDGF-AB/BB, IGF-1, V-CAM-1, and TGF-β1 presented the most elevated concentrations compared to the other molecules.

Results are described following the three parameters considered in the GLM analysis: modifications due to incubation times (1, 18, 48, 72, and 168 h), influence of the preparation type (L-PRP, P-PRP, PPP), and their combined effects (time and preparation).

GLM analysis of the concentrations of the different soluble factors examined (FGF-2, SDF-1/CXCL-12, VEGF, HGF, EGF, PDGF AB/BB, IGF-1, V-CAM-1, TGF β1, β2, and β3) (Figure 1, Figure 2, Figure 3 and Figure 4) evidenced all these factors, excluding EGF (Figure 2) and PDGF-AB/BB (Figure 3), underwent time-dependent modifications in overall preparations (*p* < 0.02 at least).

The concentrations of FGF-2 and SDF-1/CXCL-12 molecules (Figure 1) were below the detection limit of the test in several P-PRP, L-PRP, and PPP samples, mainly at the short incubation times (about 50%, 40%, and 60%, respectively), and even when increasing the incubation time, some samples remained not detectable mainly in PPP. FGF-2 showed a late significant release at 168 h (*p* < 0.01 at least).

SDF-1/CXCL-12 released after 1 h was significantly different from those at 72 and 168 h (*p* < 0.05 at least), and the concentrations detected after 18 h were different from all the following ones (*p* < 0.01 at least).

Similarly, the concentration of VEGF (Figure 2) was also below the detection limit of the test in about 30% of subjects in P-PRP and in 70% of the subjects in PPP, but it was measurable in all L-PRP samples. Lengthening the incubation time, almost all the samples became evaluable and gradually increased.

HGF concentrations (Figure 2) were similar at 1 and 18 h of incubation, and both were significantly different from all the following (*p* < 0.01 at least); the peak concentration was reached in 48–72 h, slightly increasing up to 168 h.

The lack of time-dependent modification in EGF (as evidenced by GLM analysis) (Figure 2) was mainly attributable to the stability of the concentrations from 18 h onwards. In fact, considering the preparations cumulatively, almost the whole detected EGF was released in the first 18 h with an increment of two folds at least (1 h vs. 18 h *p* < 0.02). Slight modifications were observed at the following times up to 168 h when compared with 1 h of incubation (*p* < 0.05 at least).

PDGF-AB/BB concentrations were substantially similar at all the incubation times (Figure 3).

On the contrary, IGF-1 and V-CAM-1 (Figure 3) underwent rapid releases after 1 h of incubation, and they remained highly concentrated despite the significant time-related decrease up to the 168 h. Both GFs were significantly more concentrated after 1 h and 18 h of incubation than in all subsequent incubation times (*p* < 0.05 at least).

The concentrations of TGF-β1, -β2, and -β3 similarly peaked within 48–72 h but remained stable later on (Figure 4).

In addition, GLM analysis evidenced the preparation type significantly influenced the concentrations of all soluble factors, excluding V-CAM-1 (Figure 3) and TGF-β2 (Figure 4), that were similar among the three preparations.

Another trend was observed for VEGF, HGF, EGF (Figure 2), PDGF-AB/BB, and IGF-1 (Figure 3). The preparations were significantly different from each other, being less concentrated going from L-PRP to P-PRP and PPP (*p* < 0.05 at least).

FGF-2 concentrations were similar between L-PRP and P-PRP as well as between P-PRP and PPP but were more concentrated in L-PRP than PPP (*p* < 0.01 at least) (Figure 1).

SDF-1concentrations were similar between P-PRP and PPP but were different between L- and P-PRP and between L-PRP (*p* < 0.0005) and PPP (Figure 1).

In addition, TGF-β1 and -β3 concentrations were similar between L- and P-PRP and both were higher than PPP (*p* < 0.01 at least) (Figure 4).

Finally, GLM combined analysis of time and preparation evidenced the influence of these associated parameters only for EGF, PDGF-AB/BB, and IGF-1. In particular, it was borderline in EGF (*p* = 0.061) (Figure 2) but significant in PDGF-AB/BB and IGF-1 (*p* < 0.05) (Figure 3).

Different soluble factor concentrations were positively correlated with platelet or white blood cell number, as determined by Spearman’s rank correlation coefficient (Table 2).

Most of the correlations were evident between SDF-1, VEGF, HGF, EGF, and PDGFAB/BB and both platelets and white blood cells. A fair correlation with the three isoforms of TGF-β evaluated was also evident for platelets, in particular for isoform β1 (Table 2).

### 2.3. Functional Analyses: Growth of Synovial Fibroblasts and HA Production

**Figure 5:**
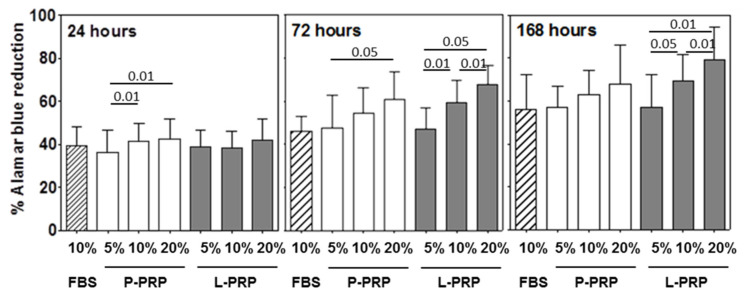
Synovial fibroblast growth following incubation with P-PRP and L-PRP. Results are reported as means and standard deviations; *p* value significances are reported, as determined by Wilcoxon matched pairs test. Differences among incubation times and PRP concentrations are reported below, as determined by Friedman Anova test. Comparisons among incubation times (24 h, 72 h, 168 h) for each concentration: FBS 10%, *p* < 0.02; P-PRP: 5% and 10%, *p* < 0.005; 20%, *p* < 0.002; L-PRP: 5%, *p* < 0.05; 10%, *p* < 0.005; 20%, *p* < 0.002. Comparisons among PRP concentrations (5%, 10%, 20%) for each incubation time: P-PRP: 24 h, 72 h, and 168 h, *p* < 0.005; L-PRP: 24 h, not significant; 72 h, *p* < 0.0005; 168 h, *p* < 0.001.

The presence of 10% FBS (used as standard control culture condition) significantly increased synovial fibroblast growth with the incubation time (Friedman Anova *p* < 0.02) (Figure 5). The increase was similar to the one induced by P and L-PRP (independently from the used percentage) up to 24 h (Figure 5); thereafter, starting from 72 h onwards, it remained lower than either P or L-PRP preparations regardless of the added percentages (Friedman Anova *p* < 0.05 at least) (Figure 5).

A progressive growth increase was induced by increasing concentrations of either P or L-PRP (Friedman Anova *p* < 0.05 and *p* < 0.005 at least, respectively) as well as by subsequent incubation times with the same amounts of P and L-PRP (Friedman Anova *p* < 0.005 and *p* < 0.05 at least, respectively). In general, the addition of L-PRP (10% or 20%) induced growth 5–10% higher than P-PRP (Figure 5).

The comparisons among L-PRP percentages showed significant progressive increases of synovial fibroblasts growth (*p* < 0.05 at least) at 72 and 168 h (Figure 5).

Control cultures without FBS run in parallel, showed a stable and comparable synovial fibroblast growth (30% Alamar Blue reduction on average) up to 168 h (Friedman Anova not significant, not shown).

Comparisons among PRP concentrations (5%, 10%, 20%) showed: P-PRP: not significant; L-PRP: not significant. Comparison between P- and L-PRP for each concentration showed: not significant.

**Figure 6:**
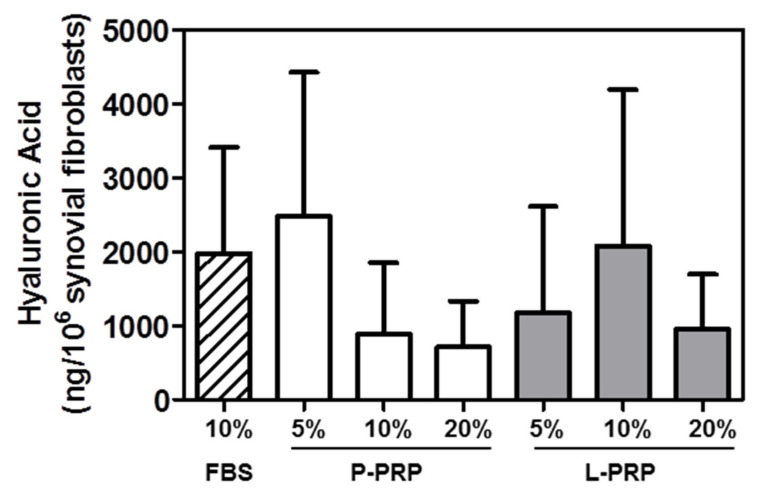
Hyaluronic acid production from synovial fibroblasts incubated with P-PRP and L-PRP. Hyaluronic acid protein production was normalized per number of synovial fibroblasts, and results are reported as means and standard deviations. Statistical analysis was performed by Wilcoxon matched pairs test and Friedman Anova test.

Synovial fibroblasts showed a conserved HA synthesis both in the presence of P and L-PRP with concentrations similar to those induced by 10% FBS and in particular a substantial stability when L-PRP was used at 5% or 10% (Figure 6).

## 3. Discussion

In recent years, PRPs are becoming common approaches of regenerative medicine in the orthopedic field, for example as a tool to treat OA. Recent meta-analyses have highlighted greater benefits deriving from PRP infiltration compared to those deriving from intra-articular treatments with corticosteroids or HA [20]. However, at present, the Osteoarthritis Research Society International (OARSI) does not include the use of PRPs among the recommended therapeutic measures [21] The study of active molecules and their release kinetics is the rationale for a sound application of platelet concentrates.

Platelet concentrates have been increasingly used to enhance tissue healing by supplying GFs at the damaged site. Articles reported on the different concentrations and release kinetics of GFs correlated with the cell characteristics of platelet preparations [14,22,23,24,25,26,27,28,29]. However, few comparative studies have been conducted on the release of GFs by L-PRP, P-PRP, and PPP (a physiological reservoir of GF) and on the ability of these different plasmas to support the growth of synovial fibroblasts and the production of HA.

In this study, we compared the time-dependent release (up to 168 h) of different GFs by different preparations of platelet concentrates as a means of providing joint tissues with pro-healing anabolic GFs and their influence on synovial fibroblast growth and HA production.

Among the various GFs present in platelet-rich plasmas, we focused on FGF-2, SDF-1, VEGF, HGF, EGF, PDGF-AB/BB, IGF-1, VCAM-1, and TGF-β isoforms and analyzed their release kinetics. A more pronounced and sustained release of GFs was observed from L-PRP than P-PRP for about 50% of the GFs evaluated; L-PRP and P-PRP releases were higher than PPP, respectively, in nine and seven out of eleven factors tested, regardless of the net concentration (picograms or nanograms).

GF release kinetics displayed three peculiar time-related patterns. IGF-1 and V-CAM-1 molecules presented the highest concentrations within 18 h and decreased afterwards, whereas FGF-2, VEGF, HGF, and EGF showed an opposite kinetic with low concentrations at the beginning of the incubation period, increasing up to 168 h. SDF-1, PDGF-AB/BB, and TGF-β isoforms exhibited an intermediate trend, reaching the highest concentrations at the central time points.

Positive correlations between platelet or white blood cell numbers and the majority of GF concentrations were also observed for each preparation.

Synovial fibroblast growth in response to L-PRP and P-PRP revealed differences over time and among added concentrations of each platelet concentrate. The most evident growth occurred in response to L-PRP at 168 h.

P- and L-PRP displayed a preserved supporting synthesis capacity of HA (a molecule playing a pivotal role in osteoarticular joint), comparable to that of FBS-supplemented synovial fibroblast cultures.

Other articles reported on some or many of the factors analyzed here and described them as involved in musculoskeletal and joint tissue homeostasis; however, to our knowledge, this is the first study that simultaneously analyzes the timing of release of these 11 factors from PRP preparations.

Independently of the kinetic characteristics, we also observed a wide individual variability, both in terms of total amount and releasing ability. Indeed, not all the factors were detectable from the beginning, but they became progressively detectable at a certain time of the evaluation period.

This aspect can be due to the late release of the bioactive content from platelets, but we cannot exclude this may be determined by the fibrin clot trapping, in agreement with a similar observation by Anitua et al. [30]. Thus, it could be argued that soluble factor concentrations became detectable in the released fraction when they exceeded the binding/retentive capacity of the fibrin scaffold. In addition, each factor could have a peculiar binding affinity to the fibrin scaffold that could reverberate on their own peculiar “ranking order” of release. This retention by the clot could affect the differential patterns of release, as supported by the data of our study, and the release rate of the cytokines that promote tissue repair when the deposition of tissue matrix starts. Therefore, the injection of PRP at weekly intervals, as scheduled in the clinical practice, does not occur after the complete metabolization of the factors. Indeed, the injection occurs in a store condition in which the seven-day growth factor concentrations still showed clearly detectable levels, not lower than the beginning ones (excluding the growth factor IGF-1 and the soluble form of V-CAM-1, which both displayed a time decreasing kinetic after the starting boost).

Different activation modalities (via CaCl_2_ or thrombin, for example) can largely influence the growth factor release from the PRP clot, in terms of both concentrations and kinetics, inducing a gradual increase or an immediate total availability of factors, respectively, as previously demonstrated [31]. The PRP activation with CaCl_2_ alone produces a blood clot that seems less structured when compared to that obtained through the addition of thrombin [32]. Furthermore, the use of CaCl_2_ alone produces a smaller PRP blood retraction, which according to Landesberg et al. [33], would be favorable to the bioavailability of GFs should they be retained for a longer period of time in the surgical bed, increasing the time of action [33,34].

We suggest both the activation method and the programmed time of administration of the PRP within the synovial fluid of the joint cavity used in clinical practice for patients with OA create a kind of “controlled environment” that prevents significant fluctuations (lack/excess) of the factors and therefore, opposite signals to the cells, thus promoting progressive tissue repair.

The immediate release of IGF-1 and V-CAM-1 could be due to simple diffusion from the plasma. The lack of GLM significance for preparations (VCAM-1) and the almost absent correlation between platelet/white blood cell numbers and both IGF-1 and V-CAM-1 GF support this reading.

In agreement with the fundamental contribution of cell components to plasma levels of the other molecules, the analysis of the secretome profile performed by using another technical approach (liquid chromatography-mass spectrometry) after three days of culture correlates with our results on EGF, PDGF, and TGF-β1 [35].

Three main limitations to consider in this study are the following.

The first is the “in vitro” model used for culturing the platelet concentrates. Obviously, the in vivo tissue environment would influence the behavior of platelet concentrates in terms of structure, cellular crosstalk, exposure to enzyme degradation, and growth factor release. The biomolecule release in “in vitro studies” measures the total accumulation over time in a solution and does not account for the denaturation, proteolysis, or the biomolecule uptake by synovial fibroblasts or for the possible additional production of other molecules. Under “in vivo” conditions, tissues’ metabolic processes can make biomolecule release differently available due to a multitude of complex interactions among tissue components. Although in vitro characterization constitutes a simplified model, it remains an important step in understanding the effects of platelets in vivo.

Second, the effect of different activators was not measured, while the influence of the different protocols cannot be ignored because the release kinetics of biomolecules could be greatly affected by the activation procedures. Furthermore, as demonstrated by our results, the PRP clot releases the various molecules differently, even though the activation factor is the same.

Third, the inter-individual variability is still a concern since basal concentration as well as the release kinetics of the biomolecules are influenced by population differences. In this connection, the impact of population differences was also observed in OA patients by Ha et al., who highlighted the variability of the composition of GFs and cytokines in PRP from patients with symptomatic knee osteoarthritis [36]. However, in the aforementioned study, seven growth factors, which we also evaluated, overlapped ours ranking of concentrations, even if these authors had used a commercial platelet concentration system and a different quantification method.

A final consideration, more general in character, concerns the need to put forward a better standardization of the laboratory procedures and clinical uses of the PRP concentrates. The study of the PRP secretome with the release times of its various constitutive macromolecules can be useful for grasping the complexity of PRP, recognizing its multifaceted therapeutic potential, and finally, moving towards a precision regenerative medicine that tailors PRP preparation to the peculiar pathological context.

## 4. Materials and Methods

### 4.1. Subjects

Ten healthy male subjects (age range 26–38 years), enrolled on a voluntary basis, underwent blood sampling. Subjects with systemic disorders, infections, hemoglobin concentrations less than 11 g/dl, platelet counts less than 150 × 103/μL, use of non-steroidal anti-inflammatory drugs within five days prior to blood collection, and smoking habits were excluded from the study. The protocol was approved by the Institutional Review Board and by the local ethic committee (Protocol N. 0008705/2014), and each donor signed an informed written consent. The samples were identified by univocal codes that guaranteed the anonymity of the subject and were appropriately processed to obtain two different PRP products.

### 4.2. Preparation of Platelet Rich Products

Two different strategies were adopted for the preparation of the PRP: a one-step centrifugation procedure, which provides a pure platelet concentrate with a limited number of platelets without leukocytes, and a two-step centrifugation procedure, which provides a concentrate with a higher platelet count containing leukocytes.

#### 4.2.1. One-Step Pure PRP (P-PRP) Method

A venous blood sample (45 mL) was collected in five tubes containing 1 mL of sodium citrate solution (3.8%) and centrifuged 8 min at 460× *g*. Then, 1 mL of the platelet-rich supernatant above the red blood cell pellet was collected from each tube, carefully avoiding underlying leukocytes [10,37,38].

#### 4.2.2. Two-Step Leukocyte PRP (L-PRP) Method

A venous blood sample (150 mL) was collected in a bag containing 21 mL sodium citrate and centrifuged 15 min at 730× *g*. The resulting plasma and buffy coat, deprived of the majority of the red blood cells, were transferred in a new bag through a closed circuit. The second centrifugation at 3800× *g* for 10 min allowed the stratification of platelet poor plasma (PPP) in the upper part and of L-PRP over the red blood cells [37]. PPP and L-PRP were separately collected.

### 4.3. Analysis of Platelet and White Blood Cell Concentrations

Platelet and white blood cell concentrations were determined by an automated hematology analyzer (Coulter LH 750, Beckman Coulter, Milano, Italy). Linearity was 5–1000 × 10^3^/μL for platelet count and 0.1–100 × 10^3^/μL for white blood cell count.

### 4.4. Activation of Platelet Concentrates

Each sample of PRP and PPP preparations was divided in five tubes (without any medium addition), activated with 10% CaCl_2_ (22.8 mM final concentration, corresponding to activation system and concentration used for clinical applications), and incubated for 1, 18, 48, 72, and 168 h at 37 °C in 5% CO_2_.

The first and the last time points were defined in agreement with the clinical practice that foresees the injection of PRP within 1 h from the CaCl_2_ activation and schedules the first three injections at weekly intervals.

At the end of each incubation time, samples were centrifuged 15 min at 2800× *g* at 20 °C, and the supernatants containing released GF were collected and frozen at −80 °C until they were tested.

### 4.5. Isolation, Culture, Growth of Synovial Fibroblasts and Hyaluronic Acid Production

Synovial fibroblasts were isolated by enzymatic digestion from patients with OA (n = 3 Kellgren–Lawrence grade II–III) undergoing joint surgery (Institutional Ethic Committee Protocol n. 8342/2010), as previously described [39]. Briefly, the synovial tissue underwent digestion with 0.1% trypsin in PBS (Sigma-Aldrich, Milan, Italy) (for 30 min at 37 °C in 5% CO2) and 0.1% collagenase P (Roche, Basel, Switzerland) (for 1 h at 37 °C) under constant rotation. The isolated cells were cultured in flasks with OPTIMEM culture medium (Gibco-BRL, Life Technologies Grand Island, NY, USA) supplemented with 100 U/mL penicillin, 100 µg/mL streptomycin (Invitrogen, Carlsbad, CA, USA) at 37 °C, and 5% CO2 in a humidified atmosphere.

Synovial fibroblasts were grown in flasks up to sub-confluence, detached, seeded in 12-well tissue-culture plates (25,000 cells/cm^2^), and then maintained with serum-free medium for 24 h. Then P-PRP, L-PRP, or PPP entire preparations just activated with CaCl_2_, as previously reported, were added to the culture medium at 5, 10, and 20% (*v*/*v*) in a Transwell device (0.4 μm porosity; Corning, Costar) inserted in the culture plates. The amount of PRP underwent clotting in the device, avoiding direct contact with synovial fibroblasts but allowing interaction with monolayers adherent to the plate bottom. Control cultures with and without 10% FBS (foetal bovine serum) were run in parallel. Plates were incubated up to 168 h without medium changes. At the end of the incubation time, Transwell devices containing clots were discarded and culture supernatants were collected and maintained at −80 °C until tested.

Cell growth and supernatant hyaluronic acid production were respectively analyzed by Alamar Blue assay (AbD Serotec, Kidlington, UK) (after 24, 72, and 168 h) and ELISA (after 168 h).

### 4.6. Supernatant Protein Evaluation

The concentration of soluble GF released from PRP preparations were determined by multiplex-beads immunoassay, as previously described [40]).

Samples were assayed in duplicate and EGF, FGF-2, HGF, IGF-1, PDGF AB/BB, SDF-1, V-CAM-1, VEGF, TGF-β1, β2, β3 concentrations were simultaneously evaluated using commercially available bead-based sandwich immunoassay kits, following the manufacturer’s instructions (Bio-Rad Laboratories, Hercules, CA, USA and Millipore Corporation, Billerica, MA, USA).

The immunocomplexes formed on distinct beads were quantified by using the Bio-Plex Protein Array System (Bio-Rad Laboratories). Data were analyzed using the Bio-Plex Manager software version 6.0 (Bio-Rad Laboratories, Hercules, CA, USA). Standard levels between 70 and 130% of the expected values were considered to be accurate and were used. In general, at least six standards were accepted and used to establish standard curves following a five-parameter logistic regression model (5 PL). Sample concentrations were immediately interpolated from the standard curves.

Values presenting a coefficient of variation beyond 10% were discarded before the final data analysis.

The concentration of HA released by synovial fibroblasts in culture supernatants was evaluated using DuoSet ELISA kit (R&D Systems) following the manufacturer’s instructions. A six-point standard curve using threefold serial dilutions and high standards (HA: 90,000 ng/mL) was performed in replicates (coefficient of variation average 18%). The accuracy of the method was assessed by evaluating the agreement between the expected and measured values by Bland–Altman plot (all differences between repeated measures and expected values did not exceed 95% confidence interval). Reliability of the test was estimated by Cronbach’s alpha coefficient (>0.99). A four-parameter logistic (4-PL) curve-fit based on standard optic density values was used to calculate the HA concentration considering three decimals.

### 4.7. Data Analysis and Statistics

Results obtained by GF dosages were analyzed by the general linear model (GLM).

Since data presented a skewed distribution not fulfilling the hypothesis of normality, appropriate log10 transformations were applied. All the resulting data fulfilled the normality assumption as verified by the Kolmogorov–Smirnov test. The GLM was used according to incubation times (1, 18, 48, 72, and 168 h), preparation types (L-PRP, P-PRP, PPP), and their association (Time*preparation) as fixed effects and patient as a random effect. Partial Eta squared (η2p) was considered as evidence of the strength of the association (effect size) between the fixed effects and concentrations of soluble factors. The Sidak correction was applied for multiple comparisons. Data were expressed as medians, interquartile ranges, and minimum/maximum values.

Cell growth results and HA concentrations, analyzed by Friedman Anova and Wilcoxon matched pair tests, were reported as means and standard deviations. Correlations between variables were determined by Spearman’s rank correlation coefficient.

A *p* < 0.05 value was considered significant.

Statistical analysis was carried out using SPSS v.19.0 (IBM Corp., Armonk, NY, USA).

## Figures and Tables

**Table 1:** Platelet-rich plasma preparations: cell concentration.

Cells	P-PRP	L-PRP	Wilcoxon Matched Pair Test
Platelets × 10^3^/μl	178 [100–343]	912 [713–995]	*p* < 0.005
White Blood Cells × 10^3^/μL	0.1 [0.1–0.2]	5.5 [4.3–7.5]	*p* < 0.005

P-PRP = pure platelet-rich plasma; L-PRP = leukocyte–platelet-rich plasma; Results are expressed as median and [interquartile ranges] of 10 donors.

**Table 2:** Correlations between growth factor concentration and platelet or white blood cell number at different incubation times.

Factors	vs. Platelet Number					vs. White Blood Cell Number				
	1 h	18 h	48 h	72 h	168 h	1 h	18 h	48 h	72 h	168 h
FGF-b										
SDF-1										
VEGF										
HGF										
EGF										
PDGF-AB/BB										
IGF-1										
V-CAM-1										
TGF-b1										
TGF-b2										
TGF-b3										

Correlations were determined by Spearman’s rank correlation coefficient: Correspondences between colors and *p* values are reported below: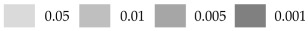.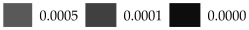.

## Data Availability

The data presented in this study will be available on request from the corresponding authors.

## Supplementary Materials

The following supporting information can be downloaded at: https://www.mdpi.com/article/10.3390/ijms24021512/s1.
